# Supplementary material for: GATA2 regulates mast cell identity and responsiveness to antigenic stimulation by promoting chromatin remodeling at super-enhancers
Source: Nat Commun. 2021 Jan 21;12:494. doi: 10.1038/s41467-020-20766-0 (PMC7820599; doi:10.1038/s41467-020-20766-0)
Supplement: Supplementary file 3 — Description of Additional Supplementary Files [file 41467_2020_20766_MOESM3_ESM.pdf]

Description of Additional Supplementary Files

Supplementary Data 1. Potential enhancers identified by H3K4me1 and H3K27ac ChIP-seq in resting mast cells, related to Fig. 1.

Supplementary Data 2. Super-enhancers and typical enhancers generated from two biological replicates, related to Fig. 1.

Supplementary Data 3. The ID genes, key ID genes, and non-ID genes in mast cells, related to Fig. 1.

Supplementary Data 4. Expression of the shared ID genes, related to Fig. 1.

Supplementary Data 5. The accessible regions located outside of the regions with H3K27ac modification in resting mast cells and with increase H3K27ac modification in activated mast cells, related to Fig. 2 and Fig. 7.

Supplemental Data 6. FPKM reads of the ID genes and the key cytokine and chemokine genes in BMMCs derived from bone marrow cells of B6 or BALB/c genetic background mice, related to Supplementary Fig. 4.

Supplementary Data 7. H3K4me1, H3K27ac ChIP-seq and ATAC-seq reads at the super-enhancers of key ID genes and RNA expression of key ID genes in WT and *Gata2* KO BMMCs, related to Fig. 3.

Supplementary Data 8. mRNA expression of the genes that have already been identified as self-renewal genes in *Gata2* WT or KO BMMCs, related to Fig. 3.

Supplementary Data 9. The complete list of RNA-seq genes and GO analysis of the induced and repressed genes in BMMCs, Related to Fig. 4. P-values were calculated by a one-side Fisher's exact test with the adjustment of Benjamini-Hochberg method.

Supplementary Data 10. Key activation-induced genes and non-key activation induced genes, related to Fig. 5.

Supplementary Data 11. H3K4me1, H3K27ac ChIP-seq and ATAC-seq reads at the typical enhancers of non-key cytokine and chemokine genes and super-enhancers of key cytokine and chemokine genes, related to Fig. 6.

Supplementary Data 12. Expression of the genes which are encoding molecules important for IgE receptor crosslinking signaling pathway in resting WT and *Gata2* KO BMMCs, related to Fig. 7.

Supplementary Data 13. Reads per enhancer for the typical and super-enhancers and RNA transcripts in resting and activated WT or *Gata2* KO BMMCs, related to Fig. 7.
