# Supplementary material for: GATA2 regulates mast cell identity and responsiveness to antigenic stimulation by promoting chromatin remodeling at super-enhancers
Source: Nat Commun. 2021 Jan 21;12:494. doi: 10.1038/s41467-020-20766-0 (PMC7820599; doi:10.1038/s41467-020-20766-0)
Supplement: Supplementary file 17 — Reporting Summary [file 41467_2020_20766_MOESM17_ESM.pdf]

nature research

Corresponding author(s): Hua Huang

Last updated by author(s): Dec 8, 2020

Reporting Summary

Nature Research wishes to improve the reproducibility of the work that we publish. This form provides structure for consistency and transparency in reporting. For further information on Nature Research policies, see Authors & Referees and the Editorial Policy Checklist .

Statistics

For all statistical analyses, confirm that the following items are present in the figure legend, table legend, main text, or Methods section.

n/a	Confirmed	
☐	☒	The exact sample size (*n*) for each experimental group/condition, given as a discrete number and unit of measurement
☐	☒	A statement on whether measurements were taken from distinct samples or whether the same sample was measured repeatedly
☐	☒	The statistical test(s) used AND whether they are one- or two-sided<br>*Only common tests should be described solely by name; describe more complex techniques in the Methods section.*
☒	☐	A description of all covariates tested
☒	☐	A description of any assumptions or corrections, such as tests of normality and adjustment for multiple comparisons
☐	☒	A full description of the statistical parameters including central tendency (e.g. means) or other basic estimates (e.g. regression coefficient) AND variation (e.g. standard deviation) or associated estimates of uncertainty (e.g. confidence intervals)
☐	☒	For null hypothesis testing, the test statistic (e.g. *F*, *t*, *r*) with confidence intervals, effect sizes, degrees of freedom and *P* value noted<br>*Give P values as exact values whenever suitable.*
☒	☐	For Bayesian analysis, information on the choice of priors and Markov chain Monte Carlo settings
☒	☐	For hierarchical and complex designs, identification of the appropriate level for tests and full reporting of outcomes
☐	☒	Estimates of effect sizes (e.g. Cohen's *d*, Pearson's *r*), indicating how they were calculated

Our web collection on statistics for biologists contains articles on many of the points above.

Software and code

Policy information about availability of computer code

Data collection	Summit (version 4.3); all sequencings were performed on an Illumina NovaSEQ6000 platform with a Paired End 150 cycle 2x150 mode.
Data analysis	Trimmomatic (version 0.33); FastQC (version 0.11.9); Bowtie2 (version 2.3.5.1); MACS2 (version 2.1.2); Integrative Genomics Viewer (IGV, version 2.8.0); STAR (version 2.4.0.1); Cufflinks (version 2.2.1); Cuffmerge (version 1.0.0); Cuffdiff (version 2.2.1); Expander software (version 7.2); DAVID (version 6.8); SAMtools (version 1.7); ROSE software (version 1); ChIPseeker (version 1.18.0); GREAT (version 4.0.4); BETA (Version 1.0.7); Bedtools (version 2.29.2); Diffbind (version 2.10.0); MEME-ChIP (version 5.2.0); ggpubr (version 0.2);deepTools (version 3.3.0); FlowJo (version 8.2).

For manuscripts utilizing custom algorithms or software that are central to the research but not yet described in published literature, software must be made available to editors/reviewers. We strongly encourage code deposition in a community repository (e.g. GitHub). See the Nature Research guidelines for submitting code & software for further information.

Data

Policy information about availability of data

All manuscripts must include a data availability statement. This statement should provide the following information, where applicable:

- Accession codes, unique identifiers, or web links for publicly available datasets
- A list of figures that have associated raw data
- A description of any restrictions on data availability

The sequencing data has been uploaded to the GEO database. And following is the detailed information about the deposited data:
Raw and analyzed data: GSE145612;
ATAC-seq data: GSE145542;
ChIP-seq data: GSE145544;
RNA-seq data: GSE145611.

Field-specific reporting

Please select the one below that is the best fit for your research. If you are not sure, read the appropriate sections before making your selection.

- [x] Life sciences
- [ ] Behavioural & social sciences
- [ ] Ecological, evolutionary & environmental sciences

For a reference copy of the document with all sections, see nature.com/documents/nr-reporting-summary-flat.pdf

Life sciences study design

All studies must disclose on these points even when the disclosure is negative.

Sample size	Tow biological samples were used for ChIP-seq analyses according to the published paper: "Initial RNA polymerase II ChIP-seq experiments showed that more than two replicates did not significantly improve site discovery (Rozowsky et al. 2009)." Two or three biological samples were used for RNA-seq analyses and two biological samples were used for Omni-ATAC-seq analyses according to the previous publications which show two or three replicates are enough for analyses.
Data exclusions	No data were excluded from the analyses.
Replication	Two or three biological replicates were used for data analyses. The reproducibility of the sequencing data was shown in supplementary figure 1 and 2.
Randomization	BMMCs derived from mice were allocated randomly into treatment groups.
Blinding	Sequencing was carried out in a blinded manner. Investigators were not blinded data analysis since we need to combine the data from same treatment for the downstream analysis.

Reporting for specific materials, systems and methods

We require information from authors about some types of materials, experimental systems and methods used in many studies. Here, indicate whether each material, system or method listed is relevant to your study. If you are not sure if a list item applies to your research, read the appropriate section before selecting a response.

Materials & experimental systems

n/a	Involved in the study	
[ ]	[x]	Antibodies
[x]	[ ]	Eukaryotic cell lines
[x]	[ ]	Palaeontology
[ ]	[x]	Animals and other organisms
[x]	[ ]	Human research participants
[x]	[ ]	Clinical data

Methods

n/a	Involved in the study	
[ ]	[x]	ChIP-seq
[ ]	[x]	Flow cytometry
[x]	[ ]	MRI-based neuroimaging

Antibodies

Antibodies used	Anti-Histone H3 (mono methyl K4) antibody-ChIP Grade (ab8895, Abcam); Anti-Histone H3 (acetyl K27) antibody - ChIP Grade (ab4729, Abcam); allophycocyanin-CY7–conjugated anti–c-Kit (2B8) (105825, Biolegend); PE-CY7–conjugated anti-FcεRIa (MAR-1) (134317, Biolegend); IgE anti-2, 4, 6-Trinitrophenyl (TNP) antibody.<br>CCL1, CCL2, CCL3, CCL4, CCL7, TNFalpha and IL-6 protein levels were detected by the ELISA kit purchased from LifeSpan BioSciences, Seattle, WA, and following are the detailed information:<br>CCL1 ELISA kit (LS-F290-1); CCL2 ELISA kit (LS-F31326-1); CCL3 ELISA kit(LS-F31325-1); CCL4 ELISA kit (LS-F31354-1); CCL7 ELISA kit (LS-F619-1); TNF alpha ELISA kit (LS-F31434-1); IL-6 ELISA kit (LS-F57505-1).
Validation	IgE anti-TNP antibody was purified from hybridoma IGEL a2 [15.3] (ATCC® TIB-142™), which was validated by our previous publication: J Allergy Clin Immunol. 2018 Oct;142(4):1173-1184.<br>All the other antibodies were validated by the manufacturers, which can be found on the following websites:<br>Anti-Histone H3 (mono methyl K4) antibody-ChIP Grade: https://www.abcam.com/histone-h3-mono-methyl-k4-antibody-chip-grade-ab8895.html;<br>Anti-Histone H3 (acetyl K27) antibody - ChIP Grade: https://www.abcam.com/histone-h3-acetyl-k27-antibody-chip-grade-ab4729.html;<br>Anti-GATA2 antibody - ChIP Grade (ab22849): https://www.abcam.com/gata2-antibody-chip-grade-ab22849.html;<br>allophycocyanin-CY7–conjugated anti–c-Kit (2B8): https://www.biolegend.com/en-us/products/apccyanine7-anti-mouse-cd117-

c-kit-antibody-5905;
PE-CY7–conjugated anti-FcεRIa (MAR-1): https://www.biolegend.com/en-us/products/pe-cy7-anti-mouse-fcepsilonrialpha-antibody-6861; 1
Mouse CCL1 ELISA Kit (LS-F290-1): https://www.lsbio.com/elisakits/mouse-i-309-ccl1-sandwich-elisa-elisa-kit-ls-f290/290;
Mouse CCL2 ELISA Kit (LS-F31326-1): https://www.lsbio.com/antibodypairs-elisadevelopmentkits/devkit-core-mouse-ccl2-mcp1-elisa-development-kit-ls-f31326/31326;
Mouse CCL3 ELISA Kit (LS-F31325-1): https://www.lsbio.com/antibodypairs-elisadevelopmentkits/devkit-core-mouse-ccl3-mip-1-alpha-elisa-development-kit-ls-f31325/31325;
Mouse CCL4 ELISA Kit (LS-F31354-1): https://www.lsbio.com/antibodypairs-elisadevelopmentkits/devkit-core-mouse-ccl4-mip-1-beta-elisa-development-kit-ls-f31354/31354?trid=247;
Mouse CCL7 ELISA Kit (LS-F619-1): https://www.lsbio.com/elisakits/mouse-ccl7-mcp3-sandwich-elisa-elisa-kit-ls-f619/619;
Mouse TNF apha ELISA Kit (LS-F31434-1): https://www.lsbio.com/antibodypairs-elisadevelopmentkits/devkit-core-mouse-tnf-alpha-elisa-development-kit-ls-f31434/31434;
Mouse IL-6 ELISA Kit (LS-F57505-1): https://www.lsbio.com/antibodypairs-elisadevelopmentkits/devkit-core-mouse-il6-interleukin-6-elisa-development-kit-ls-f57505/57505.

Animals and other organisms

Policy information about studies involving animals; ARRIVE guidelines recommended for reporting animal research

Laboratory animals

Balb/cJ (000651, the Jackson Laboratory, Bar Harbor, ME) were purchased from the Jackson Laboratory.
Inducible Gata2 knockout Gata2f/fRosaYfp/YfpTgCreErt2hemi mice or wild-type control Gata2+/+RosaYfp/YfpTgCreErt2hemi mice on 129 genetic background backcrossed to C57BL/6 genetic background for 5 generation were bred by us. The mice were generated by crossing Gata2f/f mice to Cre activity reporter RosaYfp/Yfp mice and inducible Cre mice as described before (J Allergy Clin Immunol. 2018 Oct;142(4):1173-1184).
Six-week old male or female mice were used for experiments.
Housing conditions: 72 to 74 °F ambient temperature, 50-60% humidity and 12 hours dark/light cycle.

Wild animals

The study did not involve wild animals.

Field-collected samples

The study did not involve samples collected from field.

Ethics oversight

All animal experiments were conducted according to protocols approved by the National Jewish Health Institutional Animal Care and Use Committee (Protocol number: AS2703-02-23).

Note that full information on the approval of the study protocol must also be provided in the manuscript.

ChIP-seq

Data deposition

☒ Confirm that both raw and final processed data have been deposited in a public database such as GEO.

☒ Confirm that you have deposited or provided access to graph files (e.g. BED files) for the called peaks.

Data access links
May remain private before publication.

https://www.ncbi.nlm.nih.gov/geo/query/acc.cgi?acc=GSE145612
https://www.ncbi.nlm.nih.gov/geo/query/acc.cgi?acc=GSE145542
https://www.ncbi.nlm.nih.gov/geo/query/acc.cgi?acc=GSE145544
https://www.ncbi.nlm.nih.gov/geo/query/acc.cgi?acc=GSE145611

Files in database submission

MC_UN_1_H3K4me1_treat_pileup.bdg.tdf
MC_UN_2_H3K4me1_treat_pileup.bdg.tdf
MC_ST_1_H3K4me1_treat_pileup.bdg.tdf
MC_ST_2_H3K4me1_treat_pileup.bdg.tdf
MC_UN_1_H3K27ac_treat_pileup.bdg.tdf
MC_UN_2_H3K27ac_treat_pileup.bdg.tdf
MC_ST_1_H3K27ac_treat_pileup.bdg.tdf
MC_ST_2_H3K27ac_treat_pileup.bdg.tdf
H3K4me1_WT_UN_1_treat_pileup.bdg.tdf
H3K4me1_WT_UN_2_treat_pileup.bdg.tdf
H3K4me1_WT_ST_1_treat_pileup.bdg.tdf
H3K4me1_WT_ST_2_treat_pileup.bdg.tdf
H3K4me1_KO_UN_1_treat_pileup.bdg.tdf
H3K4me1_KO_UN_2_treat_pileup.bdg.tdf
H3K4me1_KO_ST_1_treat_pileup.bdg.tdf
H3K4me1_KO_ST_2_treat_pileup.bdg.tdf
H3K27AC_WT_UN_1_treat_pileup.bdg.tdf
H3K27Ac_WT_UN_2_treat_pileup.bdg.tdf
H3K27Ac_WT_ST_1_treat_pileup.bdg.tdf
H3K27Ac_WT_ST_2_treat_pileup.bdg.tdf

H3K27Ac_KO_UN_1_treat_pileup.bdg.tdf
H3K27Ac_KO_UN_2_treat_pileup.bdg.tdf
H3K27Ac_KO_ST_1_treat_pileup.bdg.tdf
H3K27Ac_KO_ST_2_treat_pileup.bdg.tdf
MC_UN_1_H3K4me1_treat_pileup.bdg
MC_UN_2_H3K4me1_treat_pileup.bdg
MC_ST_1_H3K4me1_treat_pileup.bdg
MC_ST_2_H3K4me1_treat_pileup.bdg
MC_UN_1_H3K27ac_treat_pileup.bdg
MC_UN_2_H3K27ac_treat_pileup.bdg
MC_ST_1_H3K27ac_treat_pileup.bdg
MC_ST_2_H3K27ac_treat_pileup.bdg
H3K4me1_WT_UN_1_treat_pileup.bdg
H3K4me1_WT_UN_2_treat_pileup.bdg
H3K4me1_WT_ST_1_treat_pileup.bdg
H3K4me1_WT_ST_2_treat_pileup.bdg
H3K4me1_KO_UN_1_treat_pileup.bdg
H3K4me1_KO_UN_2_treat_pileup.bdg
H3K4me1_KO_ST_1_treat_pileup.bdg
H3K4me1_KO_ST_2_treat_pileup.bdg
H3K27AC_WT_UN_1_treat_pileup.bdg
H3K27Ac_WT_UN_2_treat_pileup.bdg
H3K27Ac_WT_ST_1_treat_pileup.bdg
H3K27Ac_WT_ST_2_treat_pileup.bdg
H3K27Ac_KO_UN_1_treat_pileup.bdg
H3K27Ac_KO_UN_2_treat_pileup.bdg
H3K27Ac_KO_ST_1_treat_pileup.bdg
H3K27Ac_KO_ST_2_treat_pileup.bdg
GATA2_UN_1_treat_pileup.bdg.tdf
GATA2_UN_2_treat_pileup.bdg.tdf
GATA2_ST_1_treat_pileup.bdg.tdf
GATA2_ST_2_treat_pileup.bdg.tdf
GATA2_UN_1_treat_pileup.bdg
GATA2_UN_2_treat_pileup.bdg
GATA2_ST_1_treat_pileup.bdg
GATA2_ST_2_treat_pileup.bdg
Gata2_WT_UN_1_ATAC_treat_pileup.bdg.tdf
Gata2_WT_UN_1_ATAC_treat_pileup.bdg
Gata2_WT_UN_2_ATAC_treat_pileup.bdg.tdf
Gata2_WT_UN_2_ATAC_treat_pileup.bdg
Gata2_WT_ST_1_ATAC_treat_pileup.bdg.tdf
Gata2_WT_ST_1_ATAC_treat_pileup.bdg
Gata2_WT_ST_2_ATAC_treat_pileup.bdg.tdf
Gata2_WT_ST_2_ATAC_treat_pileup.bdg
Gata2_KO_UN_1_ATAC_treat_pileup.bdg.tdf
Gata2_KO_UN_1_ATAC_treat_pileup.bdg
Gata2_KO_UN_2_ATAC_treat_pileup.bdg.tdf
Gata2_KO_UN_2_ATAC_treat_pileup.bdg
Gata2_KO_ST_1_ATAC_treat_pileup.bdg.tdf
Gata2_KO_ST_1_ATAC_treat_pileup.bdg
Gata2_KO_ST_2_ATAC_treat_pileup.bdg.tdf
Gata2_KO_ST_2_ATAC_treat_pileup.bdg
MC_UNvsST_gene_exp.diff
Gata2_KO_UNvsST_gene_exp.diff
Gata2_WTvsKO_UN_gene_exp.diff
Gata2_WT_UNvsST_gene_exp.diff
MC_UN_1.fpkm_tracking
MC_UN_2.fpkm_tracking
MC_UN_3.fpkm_tracking
MC_ST_1.fpkm_tracking
MC_ST_2.fpkm_tracking
MC_ST_3.fpkm_tracking
PU1.fpkm_tracking
PU2.fpkm_tracking
PS1.fpkm_tracking
PS2.fpkm_tracking
FU1.fpkm_tracking
FU2.fpkm_tracking

	FS1.fpkm_tracking<br>FS2.fpkm_tracking
Genome browser session<br>(e.g. UCSC)	https://genome.ucsc.edu/s/Yapeng%20Li/Gata2%20WT%20KO%20UN%20ST%20ChIP%2Dseq%20ATAC%2Dseq

Methodology

Replicates	Two biological replicates were used for each ChIP-seq experiment.
Sequencing depth	All sequencings were performed on an Illumina NovaSEQ6000 platform with a Paired End 150 cycle 2x150 mode.
Antibodies	Anti-Histone H3 (mono methyl K4) antibody-ChIP Grade: Abcam, ab8895;<br>Anti-Histone H3 (acetyl K27) antibody - ChIP Grade: Abcam, ab4729.<br>anti-GATA2 antibody -ChIP Grade: (ab22849, Abcam)
Peak calling parameters	The adaptor sequences in raw reads (average 24.0 million reads, two biological replicates for each group) were trimmed using Trimmomatic (version 0.33) (Bolger et al., 2014) and the quality of trimmed sequence data was analyzed by FastQC (version 0.11.9). The trimmed reads were aligned to the mm10 reference genome (GRCm38) using Bowtie2 (version 2.3.5.1) (Langmead and Salzberg, 2012). Peak calling was performed using MACS2 (version 2.1.2) with default parameters with the q-value cut-off of 0.05 (Zhang et al., 2008).
Data quality	Peak calling was performed using MACS2 (version 2.1.2) with default parameters with the q-value cut-off of 0.05 (Zhang et al., 2008).
Software	The software used for sequencing alignment and peak calling is described in Methods section ("ChIP-seq data analysis")

Flow Cytometry

Plots

Confirm that:

☒ The axis labels state the marker and fluorochrome used (e.g. CD4-FITC).

☒ The axis scales are clearly visible. Include numbers along axes only for bottom left plot of group (a 'group' is an analysis of identical markers).

☒ All plots are contour plots with outliers or pseudocolor plots.

☒ A numerical value for number of cells or percentage (with statistics) is provided.

Methodology

Sample preparation	BMMCs were cultured from bone marrow cells of mice in the presence of 20 ng/mL IL-3 as described in Methods section.
Instrument	CyAN (DakoCytomation, Glostrup, Denmark).
Software	Data collection: Summit (version 4.3);<br>Data analysis: FlowJo (version 8.2).
Cell population abundance	The purity of sorted cells was higher than 99%, which was verified by the flow cytometry analysis using the FACS sorter.
Gating strategy	BMMCs were gated using the following strategy: 1. FSLin and SSLin; 2. Live cells based on DAPI negative; 3. YFP positive cells showing the 4-OHT treatment induced Gata2 deleted or control cells; 4. FceRIa and c-Kit positive.

☒ Tick this box to confirm that a figure exemplifying the gating strategy is provided in the Supplementary Information.
